# Exploration of Noninvasive Detection of Advanced Glycation End Products in the Lens to Screen for Diabetic Kidney Disease

**DOI:** 10.3389/fendo.2022.892070

**Published:** 2022-06-29

**Authors:** Xue-Meng Zhang, Yuan Gao, Meng-Xue Yang, Xiao-Di Zheng, Rui Zhang, Yue-Yue Wu, Miao Zeng, Qian Yang, Zhi-Yan Yu, Jun Liu, Bing-Bing Zha, Bo Yang

**Affiliations:** ^1^ Department of Endocrinology, Shanghai Fifth People’s Hospital, Fudan University, Shanghai, China; ^2^ General Practice Clinic, Pujiang Community Health Service Center in Minhang District, Shanghai, China; ^3^ Center of Community-Based Health Research, Fudan University, Shanghai, China; ^4^ Department of Infectious Diseases, Shanghai Fifth People’s Hospital, Fudan University, Shanghai, China; ^5^ Department of Endocrinology, Affiliated hospital of Zunyi Medical University, Zunyi, China

**Keywords:** type 2 diabetes mellitus, diabetic nephropathy, lens, advanced glycation end products (AGEs), noninvasive technology

## Abstract

Diabetic kidney disease (DKD) is a complication of diabetes, which is the most common cause of end-stage renal disease (dialysis). DKD has a high mortality rate, and only early detection can nip this disease in the bud. Advanced glycation end products (AGEs)are generally believed to be involved in the occurrence of DKD. Studies have shown that the lens AGEs fluorescence for noninvasive detection has high consistency with the gold standard OGTT, has high sensitivity and specificity, and could be used as a practical tool for the early screening of type 2 diabetes mellitus (T2DM).Therefore, we speculated that the noninvasive lens AGEs fluorescence detection method can be used to predict the occurrence of DKD. This study detected levels of AGEs in multiple cellular and tissues and analyzed the relationships between AGEs and lens, eyeballs, peripheral blood mononuclear cell (PBMC), serum, and kidney. Additionally, we examined the possible role of lens AGEs fluorescence in DKD screening. Our preexperimental study found that lens AGE levels in patients with T2DM were positively correlated with PBM and serum AGE levels. Lens AGE levels in patients with T2DM were negatively correlated with eGFR and positively correlated with urinary ACR. The animal and cell experiments showed that the AGE levels in the eyeballs of DM mice were also positively correlated with those in the serum and kidney. To increase the reliability of the experiment, we increased the sample size. In our results, lens AGEs levels were positively correlated with the occurrence of DKD, and the incidence of DKD in the high lens AGEs group was 2.739 times that in the low lens AGEs group. The receiver operating characteristic (ROC) curves showed that patients with T2DM with a lens AGEs value ≥ 0.306 were likely to have DKD. The area under the ROC curve of the noninvasive technique for identifying DKD was 0.757 (95% Cl: 0.677-0.838, p<0.001), and the sensitivity and specificity were 70.0% and 78.7%, respectively. These results suggest that noninvasive lens AGEs detection technology has certain clinical value in diagnosing whether patients with T2DM have DKD.

## Introduction

Diabetic kidney disease (DKD) is one of the main microvascular complications of diabetes, and it is also an important cause of end-stage kidney disease (1). In China, the incidence of DKD among diabetic patients is 33.6% (2). Therefore, the early diagnosis of DKD is extremely important. Clinically, the gold standard for diagnosing various chronic kidney diseases (CKDs) is renal biopsy. However, due to subjective and objective factors, such as low acceptance by patients and the risk for complications, renal biopsy is not widely used in the clinic (3, 4).At present, noninvasive and early screening is advocated for chronic diseases, and DKD is no exception.

Studies have shown that advanced glycation end products (AGEs) can deposit in different organs (such as serum (5), blood vessels (6), the skin (7), and lens (8)et al.) and impair normal physiological functions. AGEs are directly or indirectly involved in the occurrence of DKD.

Some cells or tissues contain substances that demonstrate autofluorescence (AF), in which endogenous fluorophores that exist in human tissue emit light upon excitation by a suitable wavelength. For example, AGEs contain autofluorescent pentosides and imidazoles derived from compounds such as ketones and thus can be detected in the human body by special instruments without specific exogenous labeling. A lens fluorescence test is rapid (6 seconds) and noninvasive; it does not require fasting or any other preparation by the patient; and it is suitable for most patients who have not undergone cataract or lens replacement surgery. Furthermore, sharp objects and their associated pain, assay materials, biohazard waste, and management and disposal are eliminated. Recent studies have shown that the use of AGE for noninvasive fluorescent detection could predict the incidence of type 2 diabetes mellitus (T2DM) (9, 10).Therefore, we speculated that using AGEs in the lens can predict the occurrence of DKD.

The goal of this study was to detect levels of AGEs in multiple cells and tissues, observe the relationship between AGEs and DKD from multiple angles, and assess whether the use of noninvasive lens AGE can predict the development of DKD in T2DM. Further establishing a noninvasive technique to identify DKD will lay the foundation for future research on models to predict the risk for DKD.

## Study Design and Methods

### Study Objects

#### Study Population Grouping, Inclusion Criteria and Sample Acquisition

(1)Preexperimental A total of 104 patients with T2DM diagnosed at the Department of Endocrinology within Shanghai Fifth People’s Hospital Affiliated to Fudan University from September 2019 to May 2020 were included in the first part of the study. The patients were divided into 2 groups according to the presence or absence of DKD: the patients without DKD were the control group (69 patients: 37 males and 32 females), and those with DKD were the DKD group (35 patients: 19 males and 16 females).

(2)Based on the preexperimental study, we increased the sample size. A total of 2810 patients with T2DM diagnosed at the Shanghai Pujiang Community Health Service Center and Shanghai Fifth People’s Hospital were chosen from September 2019 to March 2021. Patients who did not meet the inclusion criteria or who demonstrated poor compliance were excluded. Finally, a total of 256 patients with T2DM (155 males and 101 females) were included in the third part of the study. The patients were divided into a non-DKD group (171 patients: 107 males and 64 females)and a DKD group (85 patients: 48 males and 37 females).

(3) Inclusion and exclusion criteria for all subjects: The inclusion criteria for the T2DM group, which were based on the 2017 Chinese Medical Association Diabetes Association “Chinese T2DM Prevention and Treatment Guidelines” (11), were as follows: urine albumin-to-creatinine ratio (ACR) of <30 mg/g; urinary microalbumin (UALB)<30 mg/24 h. The inclusion criteria for the DKD group were based on the 2021 Chinese Medical Association Nephrology Branch “Chinese Guidelines for Clinical Diagnosis and Treatment of Diabetic Kidney Disease” (12) and were as follows:①met the diagnostic criteria for T2DM;②had severe albuminuria (urine ACR≥300 mg/g, UALB ≥300mg/24h), diabetic retinopathy with microalbuminuria (urine ACR: 30-300 mg/g, or UALB: 30-300 mg/24h) and/or an estimated glomerular filtration rate (eGFR)<60 min/ml. The exclusion criteria were as follows:①younger than20 years or older than 70 years of age;②had gestational diabetes or other special forms of diabetes;③had acute and chronic infections, benign and malignant tumors, blood system diseases, immune system diseases, urinary calculi, CKD without DKD, or DKD combined with non-DKD;④underwent a fluorescence angiogram within the past 6 months;⑤underwent treatment with photodynamic drugs within the past year;⑥clinically diagnosed with cataracts in the tested eye or had any signs of opacification;⑦had ocular surface disease (dry eye);⑧demonstrated poor compliance”.

(4) The procedures were approved by the Ethics Board at Shanghai Fifth People’s Hospital, Fudan University(approval no. 2021-142).Study participants with a clinical diagnosis of T2DM or DKD provided informed consent. The clinical characteristics of the study participants, including sex, age, and BMI, were collected (see **Tables 1**, **2**).

**Table 1:** PCR primer sequences.

Gene	Ipstream primer(5′–3′)	Downstream primer(3′–5′)
TLR4	ATGGCATGGCTTACACCACC	GAGGCCAATTTTGTCTCCACA
NF-κB	AGGCTTCTGGGCCTTATGTG	TGCTTCTCTCGCCAGGAATAC
β-actin	GGCTGTATTCCCCTCCATCG	CCAGTTGGTAACAATGCCATGT

**Table 2:** Comparison of general data and biochemical indexes of T2DM patients.

	non-DKD	DKD	Pvalue
Male/Female (n)	37/32	19/16	
Age (y)	65.5 ± 2.5	65.4 ± 2.8	0.763
BMI (kg/m^2^)	24.7 (22.8,26.7)	25.8 (21.9,27.4)	0.778
FBG (mmol/L)	7.2 ± 2.4	9.3 ± 5.8	**0.042**
2hPBG (mmol/L)	11.9 ± 4.5	12.1 ± 4.5	0.857
Insulin (pmol/ml)	49.2 (25.9,66.9)	47.4 (14.8,81.3)	0.509
2h-Insulin (pmol/ml)	201.3 (74.4,371.4)	135.7 (72.0,223.5)	0.079
FC-P (nmol/L)	0.63 (0.39,0.88)	0.59 (0.31,1.00)	0.970
2hC-P (nmol/L)	1.44 (0.70,2.07)	1.15 (0.54,1.99)	0.102
HbA1c (%)	8.2 (7.3,9.7)	9.5 (7.2,10.0)	**0.003**
FRA (umol/L)	361 (313,453)	381 (310,453)	0.099
eGFR (ml/min/1.73m^2^)	99.0 ± 18.4	88.4 ± 37.5	**0.019**
urinary ACR (mg/g)	9 (4,11)	78 (37,498)	**<0.001**
lens AGEs	0.256 ± 0.055	0.335 ± 0.080	**<0.001**
SerumAGEs (μg/ml)	28.49 ± 3.15	42.67 ± 5.18	**<0.001**
AGEs in PBMC	0.9 (0.8,1.1)	2.7 (2.0,3.2)	**<0.001**

non-DKD is Non diabetic kidney disease group; DKD is diabetic kidney disease group; BMI is weight (kg)/height (m^2^);FBG is fasting blood glucose;2hPBG is 2-hour postprandial blood glucose; FC-P is fasting C-peptide; 2hC-P is C-peptide 2 h after a meal; FRA is fructosamine; eGFR is estimated glomerular filtration rate; urinary ACR is the ratio of urinary microalbumin to creatinine;PBMC is peripheral blood mononuclear cell. The bold values means that the value of P was less 0.05 or 0.001, which was significant.

#### Study Mice and Grouping

Sixty KKaymice with spontaneous T2DM and 30 C57BL/6 mice [Beijing Huafukang Biotechnology Co., Ltd. (License No.: SCXK (Jing) 2020-0004))] were used for the second part of the study. All mice were 8-week-old males, weighed between 18 and 22 g and were raised in an SPF animal room with appropriate room temperature, humidity and indoor light and dark alternation times.

Grouping: DM group (n=30) and DKD group (n=30), C57BL/6 mice as control group (n=30).

This study was reviewed by the Animal Ethics Committee of the Affiliated Hospital of Zunyi Medical University.

#### Podocyte Culture and Grouping

The mouse glomerular podocyte cell line (MPC5) was donated by Professor RuanXiongzhong of the Royal Public Hospital of England. The resuscitated MPC5 cell line was inoculated into a 25-cm^2^culture flask coated with type I collagen (Sigma, USA) and cultured at 33°C under the induction of 10 U/ml recombinant mouse interferon-gamma (Sigma, USA) to 85% confluency. The cells were detached with 0.05% trypsin-EDTA (Gibco company) and then passaged; subsequently, 10% FBS (HyClone company) RPMI1640 (Gibco company) medium was added, and the cells were cultured at a density of2x10^5^/flask or2x10^3^/well. The cells were cultured in a 25-well culture flask coated with type I collagen in a 5% CO_2_ incubator for 8-10 days at 37°C without γ-interferon for differentiation, and the medium was changed every 2 days.

Grouping: Control group (n=6), DM group (n=6) and DM+AGEs group (n=6).

Control group (incubated with 5.5 mmol/L glucose), DM group(incubated with 22.2 mmol/L glucose) and DKD group (incubated with 22.2 mmol/L glucose and 0.2 mg/ml AGEs).The corresponding concentrations of glucose and AGEs were added to the above groups for 48 hours, and each experiment was replicated 6 times. The selection of the concentrations of glucose (Sigma, USA) and AGEs (Sigma, USA) was based on reference (13), the previous National Natural Science Foundation of China (grant number 81560147), and the results of the preexperiment.

### Detailed Research Method

#### Biochemical Index Detection

A Sysmex XN-9000 automatic blood cell analyzer was used to perform routine blood tests [white blood cell (WBC), neutrophil, monocyte, lymphocyte, red blood cell(RBC), hemoglobin (HGB) and platelet (PLT) analyses]. An American Bole D-100 glycosylated hemoglobin analyzer was used to detect glycosylated hemoglobin (HbA1c). A Roche Cobas 8000 automatic biochemical analyzer was used to detect fasting blood glucose (FBG), 2-h postprandial blood glucose (PBG), fructose mine (FRA), serum creatinine (Scr), trace albumin protein in the urine (UALB), and urine creatinine (UCR). A Roche Cobase 601 automatic electrochemiluminescence immunoassay analyzer was used to detect fasting C-peptide (FC-P) and C-peptide (PC-P) levels 2 h after a meal.

#### AGEscan Fluorescence Detection

A qualified medical staff member who had received standardized training noninvasively evaluated the research subjects with a lens AGEscan fluorescence detector (provided by Sannuo Biosensing Co., Ltd.). The detector emitted blue light through a blue LED and illuminated the lens of the patients’ left eye, exciting the AGEs in the lens and producing fluorescence. The detector then measured this fluorescence signal. Because there is a positive correlation between fluorescence intensity and AGEs, we could thus determine the accumulation level of AGEs.

#### KK Mouse Breeding

Thirty-six KKay mice were divided into the DM group (n=18, reared until 14 weeks of age) and the DKD group (n=18, reared until 20 weeks of age). C57BL/6 mice were used as the control group (n=18, reared to 14 weeks of age).The DM group was defined as tail vein blood glucose measurements higher than 16.7 mmol/L 3 consecutive times on different days (14), and the DKD group had 24-hour urinary protein excretion ≥30 mg or urine ACR ≥30 mg/g (15).Urine, serum, eyeballs and kidneys were collected from the mice for subsequent experiments.

#### Peripheral Blood Mononuclear Cell Isolation

Six milliliters of peripheral blood was taken from the research subjects and anticoagulated with heparin sodium. The mononuclear cell suspension was obtained by Ficoll density gradient centrifugation and washed 2-3 times with phosphate-buffered saline (PBS) to prepare a cell suspension with a concentration of 3x10^6^ cells/ml. The cells were then stained with trypan blue, and the cell viability was > 95%.

#### Liquid-Based Thin-Layer Cytology Combined With Indirect Immunofluorescence Staining to Observe the Expression of Podocin in Podocytes in Urine

The supernatant was discarded, and the lower layer of the cell pellet was retained. Fixative was then added, and PBS was used to wash the samples 2-3 times. The samples were subsequently centrifuged, and the supernatant was discarded. The samples were then shaken well and placed in Prep StatinTM automatic liquid-based cell solution for imaging. Then, indirect immunofluorescence staining was used to detect the expression of podocin in podocytes.

#### Western Blotting

Total protein from cells or tissues was harvested with ice-cold RIPA buffer (10 mM Tris, 150 mM sodium chloride, 0.5% sodium deoxycholate, 1% Triton X-100, 0.1% SDS, 1% NP-40, pH 7.4) plus protease inhibitor cocktail (Thermo Fisher Scientific) and phosphatase inhibitor cocktail (Roche). Equivalent amounts of protein were separated by 10% or 12% SDS–PAGE and transferred onto a 0.22-mm PVDF membrane (Merck Millipore, Burlington, MA, USA). The membranes were blocked with 5% skimmed milk (w/v) in TBST buffer for 1 h and incubated with the rabbit polyclonal to AGE antibody (Abcam, 1:1000) with gentle rocking overnight at 4°C. The membranes were further incubated with goat anti-rabbit HRP-conjugated secondary antibodies (Cell Signaling Technology, 1:1500) for 1.5 h at room temperature. Finally, protein bands were imaged using an enhanced chemiluminescence detection kit (Pierce ECL Plus, Thermo Scientific) and analyzed with a luminescent image analyzer (ImageQuant LAS 4000 or Amersham Imager 600, GE Healthcare Life Sciences).

#### Quantitative Real‐Time Polymerase Chain Reaction

RNA isolation and RT–qPCR: Total RNA was extracted from frozen tissues or cultured cells using TRIzol reagent (Invitrogen, Carlsbad, CA, USA), subjected to reverse transcription and analyzed by RT–qPCR using commercial kits (Vazyme Biotech, Nanjing, China). Reactions were conducted in the 384-well format with a LightCycler 480 II Instrument (Roche Diagnostics, Mannheim, Germany), and relative mRNA abundance was normalized to ribosomal 18S RNA or cyclophilin A using the 2-DDCt method. The primer sequences used are listed in **Table 1**.

#### HE, Masson Staining and PAS Taining to Observe Pathological Changes in Kidney Tissue

(1) HE staining: Kidney tissue was fixed with 4% paraformaldehyde solution and embedded in paraffin for sectioning. After staining with hematoxylin solution for 3-5 min, the sections were washed with 1% acidic ethanol (1 ml of hydrochloric acid, 99 ml of 70% ethanol), rinsed with running water, stained with eosin solution for 0.5-1 min, dehydrated, mounted, and placed under a light microscope (200×) for observation and imaging.

(2) Masson staining: The sections were dewaxed and placed in potassium dichromate overnight, followed by hematoxylin, Ponceau red acid fuchsin, phosphomolybdic acid, and aniline blue staining, dehydration, mounting, and imaging under a light microscope (200x).

(3) PAS staining Paraffin sections were deparaffinized to water, rinsed with distilled water, washed with 70% alcohol, immersed in periodic acid for acidification, washed with pure water, stained with Schiff’s solution in the dark, washed with running water, stained with hematoxylin counterstain, and differentiated with alcohol, after washing with pure water, dehydrated coverslips, and imaging under a light microscope (400x).

#### Immunofluorescence Observation of Podocin Expression in the Kidney and Podocytes

(1) Mouse kidney immunofluorescence: Immunofluorescence analysis of 4% paraformaldehyde-fixed, 0.2% Triton X-100-permeabilized frozen sectioned mouse kidney tissue labeled with podocin at a 1/100 dilution, followed by rabbit monoclonal primary antibody (Abcam, U.K) at a 1/1000 dilution, was performed. The secondary antibody was anti-rabbit IgG(Santa Cruz Company, USA) at a 1/1000 dilution. The nuclear counterstain was DAPI. Fluorescent images (300msexposure time) were obtained with an AxioImager. Z2 microscope (Carl Zeiss, Germany). The kidneys were photographed with a fluorescence microscope. Each section was observed and photographed under a fluorescence microscope (400x).

(2) Podocyte immunofluorescence: After treatment, the cells were washed with PBS, fixed with 4% paraformaldehyde, blocked with bovine serum, permeabilized with 0.5% Triton X-100 for 10 minutes, incubated with rabbit monoclonal primary antibody(Abcam, U.K) at a 1:400 dilution overnight, incubated with anti-rabbit IgG secondary antibody (Santa Cruz Company, USA)at a 1:1000 dilution overnight for 1 hour, and washed with PBS. The nuclear counterstain was DAPI. mounted with anti-fluorescence quenched mounting medium and imaged with a fluorescence microscope. Each section was observed and imaged under a fluorescence microscope (400x).

#### Observation of Renal Pathological Changes Under an Electron Microscope

##### Harvest of Tissue, Blocking and Fixation

The 1mm^3^ tissue blocks were transferred into an EP tube with fresh TEM fixative for further fixation and stored at 4°C for preservation and transportation. Then, the tissues were washed using 0.1 M PB (pH 7.4) 3 times for 15 min each.

##### Postfixation

Tissues were fixed in the dark with 1% OsO4 in 0.1 M PB (pH 7.4) for 2 h at room temperature. OsO4 was then removed, and the tissues were rinsed in 0.1 M PB (pH 7.4) 3 times for 15 min each.

##### Dehydration at Room Temperature

The tissues were incubated in 30% ethanol for 20 min;50% ethanol for 20 min;70% ethanol for 20 min;80% ethanol for 20 min;95% ethanol for 20 min; twice in 100% ethanol for 20 min; and finally twice in acetone for 15 min.

##### Resin Penetration and Embedding, Polymerization and Ultrathin Sectioning Were Performed

##### Staining

The sections were stained with 2% uranium acetate saturated alcohol solution in the dark for 8 min, rinsed in 70% ethanol 3 times and then rinsed in ultrapure water 3 times. Then, sections were stained with 2.6% lead citrate in the absence of CO_2_ for 8 min and rinsed with ultrapure water 3 times. After drying with filter paper, the cuprum grids containing the sections were placed onto the grid board and dried overnight at room temperature.

##### Observation and Image Capture

The cuprum grids were observed under TEM, and images were taken.

### Statistical Methods

All data were analyzed using SPSS 25.0 statistical software. The Shapiro–Wilk test was used to analyze whether the data had a normal distribution. Normally distributed data are expressed as the mean ± standard deviation ( x± s), and the median interquartile range [M(P25, P75)] was used for nonnormally distributed data. The independent samples t-test and Kruskal–Wallis one-way ANOVA were used to compare normal and nonnormal distributions between groups. Correlation analysis was performed using Pearson’s correlation test. Logistic regression analysis was used to evaluate independent risk factors, and the receiver operating characteristic (ROC) curve was used to evaluate the sensitivity and specificity of noninvasive AGEs for identifying DKD risk. P<0.05 was considered statistically significant.

## Results

### Lens AGEs in T2DM Patients Were Positively Correlated With PBMCs and Serum AGEs

**Figure 1:**
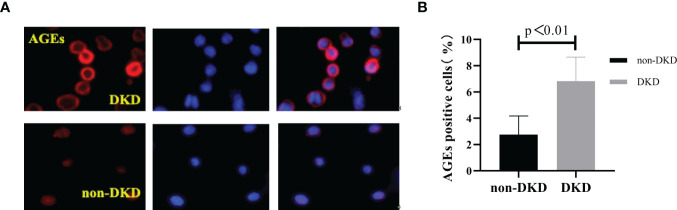
The number of age-positive cells in PBMCs of T2DM patients with DKD was higher than that of T2DM patients without DKD. **(A)** Immunofluorescence; Red: the age-positive cells; Blue: DAPI staining the nucleus. **(B)** The comparison of age-positive cells in PBMCs of T2DM patients with DKD and that of T2DM patients without DKD.

**Figure 2:**
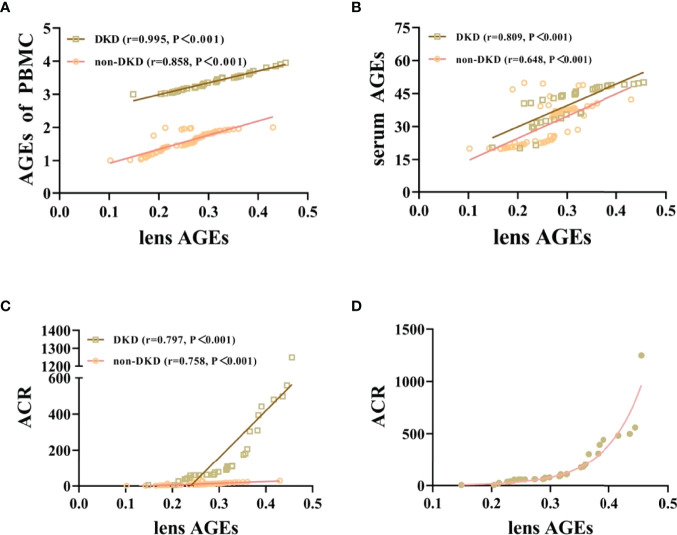
Lens AGEs in patients with T2DM were positively correlated with PBMCs and serum AGEs and urinary ACR. **(A)** Correlation analysis of lens AGEs and PBMC AGEs in patients with T2DM (400x). **(B)** Correlation analysis of lens AGEs and serum AGEs in patients with T2DM. **(C)** Correlation analysis of lens AGEs and urinary ACR in patients with T2DM. **(D)** In the DKD group, the detection value of lens AGEs gradually increased with increasing urinary ACR.

**Figure 3:**
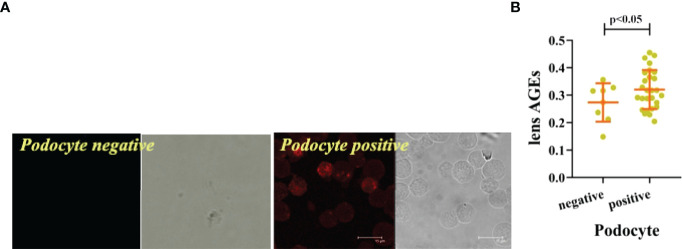
The lens AGEs in the podocyte-positive group of DKD patients were higher than those in the podocyte-negative group. **(A)** The lens AGEs in the podocyte-positive group of DKD patients were higher than those in the podocyte-negative group (n = 27 in the podocyte-positive group; n = 8in the podocyte-negative group). **(B)** Comparison of lens AGEs between the podocyte-positive group and the podocyte-negative group (n = 27 in the podocyte-positive group; n = 8 in the podocyte-negative group).

We collected peripheral blood PBMCs and serum from 104 T2DM patients with DKD (n=35) and without DKD (non-DKD) (n=69) and measured lens, PBMCs and serum AGEs. We found that the expression levels of lens, PBMC and serum AGEs in T2DM patients with DKD were higher than those in T2DM patients without DKD (see **Table 2** and **Figure 1**).Pearson’s correlation analysis showed that lens AGEs were positively correlated with PBMCs, serum AGEs and urinary ACR in both the DKD and non-DKD groups (see **Figures 2A–C**).We also found that lens AGEs levels in the DKD group gradually increased with increasing urinary ACR (see **Figure 2D**) (p < 0.05). A total of 27 podocyte-positive cases were detected by the urine TCT method of indirect immunofluorescence staining. The podocyte positivity rate was 77.1% (27/35). Patients were divided into two groups based on podocyte positive and negative status, and the difference between the lens AGEs level and the urine ACR was compared between the two groups. We found that the lens AGEs level of the podocyte-positive urine group (0.32 ± 0.07) was significantly higher than that of the podocyte-negative urine group (0.27 ± 0.07) (p< 0.05)(see **Figure 3**). This finding further confirms that AGE levels are closely related to the occurrence and development of DKD.

Three sections were observed per specimen, 10 high-power fields were randomly selected for counting, and ImageJ software was used to count the number of AGE-positive cells. The results showed that the number of AGE-positive cells in PBMCs of T2DM patients with DKD was significantly higher than that of T2DM patients without DKD (p<0.05) (**Figure 1**).

### Eyeball AGEs in DKD Mice Were Positively Correlated With Kidney and Serum AGEs

**Figure 4:**
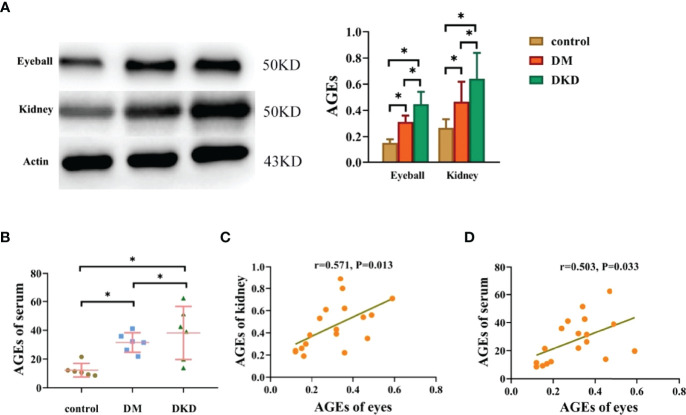
Eyeball AGEs in DKD mice were positively correlated with kidney and serum AGEs (*p<0.01). **(A)** Representative pictures of eyeball and kidney AGEs in mice in the control group, DM group and DKD group (n =6 mice per group). **(B)** Representative pictures of serum AGEs in mice in the control group, DM group and DKD group(n =6 mice per group). **(C)** Correlation analysis of AGEs in the eyes and kidneys of mice (n =6 mice per group). **(D)** Correlation analysis of AGEs between the eyes and serum (n =6 mice per group).

To verify the relationship between AGEs in different tissues and organs *in vivo*, we constructed DM and DKD mouse models to observe the expression of AGEs in the eyeball, kidney and serum. The protein expression of AGEs in the eyeball and serum of the DKD group was significantly higher than that of the DM group and the control group (see **Figure 4A**), and the expression of AGEs in the eyeballs of DM mice was positively correlated with both serum AGEs and kidney AGEs (see **Figures 4B, C**).These data suggest that AGEs are deposited in different tissues and that there is a certain correlation between AGE levels in different tissues. Previous studies have confirmed that the deposition of AGEs in different tissues affects the pathological changes in normal tissues, including the kidney and lens (16–18).These results, combined with those obtained with previous human experiments, indicate that noninvasive lens AGEs level detection is likely a fast method for predicting the risk for DKD.

### AGEs Promote Renal Pathological Damage and Podocyte Injury in DKD Mice Through the NF-κB Signaling Pathway

qPCR was used to detect the mRNA expression levels of NF-κB in kidney tissue. Compared with those in the control group, the mRNA expression levels of

**Figure 5:**
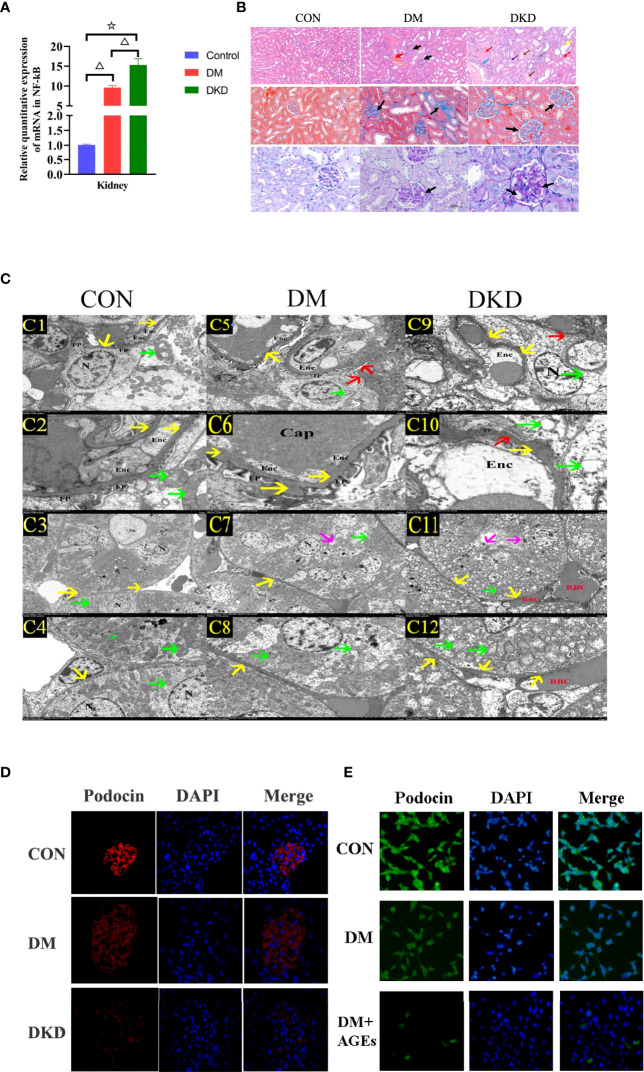
AGEs promote pathological renal damage in DKD mice through the NF-κB signaling pathway. **(A)** Relative expression of NF-κB mRNA in renal tissues of three groups of mice(n = 6 mice per group). (^△^P<0.01 vs. control;^☆^P<0.01 vs. DM.). **(B)** Representative pictures of HE, PAS and Masson’s trichrome staining in mice from the control group, DM group and DKD group(n = 6 mice per group). **(C)** Representative pictures of electron microscopy in mice from the DKD group (C1–C4),DM group (C5-C8) and control group (C9 and C12) (n = 6 mice per group). C1, C5, C9 (2.5 K), C2, C6, C10 (8.0 K), C3, C7, C11 (1.0 K), C4、C8、C12 (2.5K);C1、C2、C5、C6、C9、C10: glomerular podocyte;C3、C4、C7、C8、C11、C12:proximal tubule epithelial cell; Enc: Endothelial cells, RBC red blood cells, FP foot process, N nucleus, yello warrow basement membrane, red arrow the foot process (FP) was fused and widened, green arrow mitochondria, magenta arrow the cell membrane was damaged. **(D)** Representative images of immunofluorescence in mouse podocytes from the control group, DM group and DKD group(n = 6 mice per group) (400x). **(E)** Representative images of immunofluorescence in mouse podocytes from the control group, DM group and DM+AGEs group(n = 6 mice per group)(400x). Podocytes were incubated with 0.2 mg/ml AGEs for 48 hours.

NF-κB in the kidney tissue of the DM and DKD groups was upregulated; NF-κB expression in the DKD group was higher than that in the DM group, and the difference was statistically significant (all P< 0.05) (see **Figure 5A**).In the DM group, a small amount of glomerular stroma and mild connective tissue hyperplasia can be seen in the interstitium, and blood vessels and capillaries are congested. In the DKD group, the volume of glomeruli is enlarged, and the matrix is ​​hyperplastic. There are swollen renal tubular epithelial cells, loose cytoplasm and light staining, which can be seen locally accompanied by renal tubular dilatation and atrophy. In interstitial tissue, there is vascular and capillary congestion. And there are obvious renal interstitial fibrosis changes in masson staining and glomerulus shows focal segmental glomerulosclerosis (FSGS), thickening of capillary walls and microangioma in PAS staining(arrows)(see **Figure 5B**). According to electron microscopy scanning, for proximal tubule epithelial cells, we can see that in the DKD group, the epithelial cells in the proximal tubules are markedly edematous, with extensive damage to the cell membrane, local blurring, and local low electron density edema in the cells. The number of mitochondria is abundant, most of which are obviously swollen and slightly enlarged, the intramembrane matrix became lighter, and the cristae is broken and reduced; the red blood cells (RBCs) in the renal interstitium are massively aggregated, resulting in renal congestion. For glomerular podocytes, we can see that the glomerular podocytes show moderate edema, and the cell membrane is partially damaged, with a reduced electron density of the intracellular matrix; the organelles are abundant, and the swelling and vacuolation are obvious. Mitochondria are significantly swollen, the intramembrane matrix is dissolved, the cristae was reduced and disappeared, and the vacuoles are changed; the foot processes (FP) are partially fused and significantly widened; and the basement membrane (BM) is significantly thickened and bulged locally. Vascular endothelial cells show severe edema (see **Figure 5C**).Podocin is a podocyte-specific gene. The glomerular volume of DM and DKD mice increases, and podocin expression is attenuated (see **Figure 5D**). Podocin expression in podocytes is downregulated after incubation with AGEs in high glucose conditions (see **Figure 5E**).

### Noninvasive Lens AGEs Level Measurement Has Good Clinical Application Value for Predicting DKD Risk in Patients With T2DM

**Figure 6:**
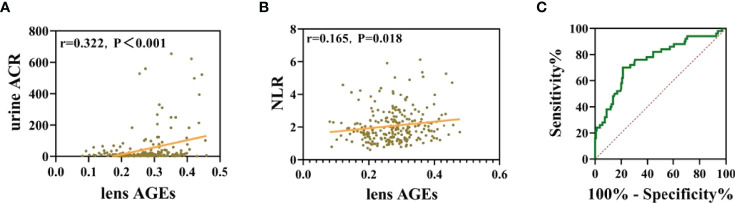
Noninvasive lens AGE measurement has good clinical application value for predicting DKD risk in patients with T2DM. **(A)** Lens AGE levels were positively correlated with urinary ACR (n = 256). **(B)** Lens AGE levels were positively correlated with NLR (n = 256). **(C)** ROC curve analysis showed that lens AGE levels were an independent risk factor for DKD.

Patients who did not meet the research criteria or demonstrated poor compliance were excluded. A total of 256patients with T2DMwere included, 171 without DKD (107 males and 64 females) and 85 with DKD(48 males and 37 females) (see **Table 3** and **Figure 6A**).

**Table 3:** Paired case-control study of non-DKD and DKD.

	non-DKD	DKD	p
	n=171	n=85	value
Gender			0.348
Male	107 (62.6%)	48 (56.5%)	
Female	64 (37.4%)	37 (43.5%)	
Age (y)	59 (50~65)	60.5 (51~66)	0.582
BMI (kg/m^2^)	24.80 ± 4.08	24.63 ± 4.11	0.754
FBG (mmol/L)	7.20 (5.44~9.53)	8.24 (6.20~11.15)	**0.009**
2hPBG (mmol/L)	12.27 (9.45~15.40)	13.18 (8.27~16.80)	0.223
HbA1c (%)	8.4 (7.2~10.2)	9.6 (8.0~11.2)	**<0.001**
FRA (umol/L)	396.335 ± 110.814	428.165 ± 116.229	0.077
Insulin (pmol/ml)	16.32 (24.05~72.15)	55.46 (27.20~78.91)	0.641
2h-Insulin (pmol/ml)	279.818 ± 291.583	227.088 ± 276.336	0.251
FC-P (nmol/L)	0.65 ± 0.40	0.70 ± 0.46	0.410
PC-P (nmol/L)	1.65 ± 1.19	1.34 ± 1.16	0.070
CysC (mg/L)	0.90 ± 0.18	1.08 ± 0.22	**0.003**
25 (OH)D (nmol/L)	46.65 ± 17.32	42.44 ± 16.26	0.082
N-MID (ng/ml)	11.74 (9.78~16.27)	9.69 (7.47~12.72)	0.073
β-CTX (pg/ml)	436.2 ± 234.4	417.4 ± 318.1	0.642
eGFR (ml/min/1.73m^2^)	106 (89~121)	93 (59~125)	**0.011**
ACR (mg/g)	8.67 ± 6.31	345.18 ± 850.74	**<0.001**
WBCs (*10^9^/L)	6.00 ± 1.73	6.47 ± 1.73	**0.043**
Neutrophils (*10^9^/L)	3.00 (2.34~4.18)	4.10 (3.13~5.86)	**0.006**
Monocytes (*10^9^/L)	0.47 ± 0.18	0.51 ± 0.17	0.075
Lymphocyte (*10^9^/L)	1.90 ± 0.66	1.90 ± 0.69	0.885
RBCs (*10^12^/L)	4.55 (4.35~4.87)	4.29 (4.17~4.94)	0.347
HGB (g/L)	141 (128~150)	132 (126~145)	0.113
PLTs (*10^9^/L)	205 (168~240)	227 (178~274)	**0.016**
NLR	1.956 ± 0.915	2.328 ± 1.142	**0.013**
Detected value of lnoninvasive lens AGEs	0.270 (0.214~0.298)	0.320 (0.287~0.386)	**<0.001**

non-DKD is Non diabetic kidney disease group;DKD is diabetic kidney disease group; BMI is weight (kg)/height (m^2^);FBG is fasting blood glucose;2hPBG is 2-hour postprandial blood glucose; FRA is fructosamine; FC-P is fasting C-peptide; 2hC-P is C-peptide 2 h after a meal; eGFR is estimated glomerular filtration rate;urinary ACR is the ratio of urinary microalbumin to creatinine; WBCs is white blood cells; HGB is hemoglobin; PLTs is platelets; NLR is neutrophils and lymphocytes ratio. The bold values means that the value of P was less 0.05 or 0.001, which was significant.

Pearson correlation tests were used to analyze the correlations among lens AGEs levels, eGFR, Urine ACR and NLR. The results showed that lens AGEs levels were positively correlated with urine ACR and NLR(r=0.322, p<0.001 and r=0.165, p=0.018) (see **Figures 6B, C**).

Through binary logistic regression analysis, we found that the incidence of DKD in the high lens AGEs group was 2.739 times that in the low lens AGEs group. The receiver operating characteristic (ROC) curves showed that patients with T2DM with a lens AGEs value ≥ 0.306 were likely to have DKD. The area under the ROC curve of the noninvasive technique for identifying DKD was 0.757 (95% Cl: 0.677-0.838, p<0.001), and the sensitivity and specificity were 70.0% and 78.7%, respectively (see **Figure 6B, C**). In summary, the level of lens AGEs can be used as an independent risk factor for the diagnosis of DKD.

## Discussion

Statistically, approximately 31.3% of all new end-stage renal disease (ESRD) worldwide in 2015 was caused by diabetes mellitus (DM) (19).The gold standard for CKD diagnosis is renal biopsy, but its low acceptance rate and high economic cost limit its application in the clinic. The diagnosis of DKD is no exception. According to the CDS guidelines, patients with DM are assessed for DKD with urine ACR and eGFR analyses, but not all DM patients can receive invasive or complex tests to screen for DM-related kidney damage. Therefore, a more convenient, easy-to-operate, noninvasive technology is required for DKD screening in the DM population for early identification and evaluation purposes.

French chemist L.C. Mailard first reported advanced glycation end products (AGEs) in 1912, and they were discovered when amino acids and glucose were mixed and heated. AGEs are a general term for a class of stable end products formed after a series of reactions between the free amino groups of proteins and amino acids and the carbonyl groups of reducing sugars. AGEs are not easily degraded. Studies have shown that AGEs play an important role in the occurrence and progression of DKD (20–24). It damages kidney cells through RAGE activation by binding to its receptor, thereby leading to the onset of DKD (20, 23).We found that lens AGE levels in patients with T2DMwere positively correlated with PBMC and serum AGE levels. It was also positively correlated with urinary ACR and negatively correlated with eGFR. Our study showed that the expression of eyeball AGEs in DM mice was also positively correlated with AGE levels in the serum and kidneys. This is similar to the results of previous studies (9).We also found that AGEs promoted renal pathological damage and podocyte injury in DKD mice through the NF-κB signaling pathway, which was consistent with the results of Coughlan MT et al (21, 25). The characteristics of AGEs deposited in multiple tissues and cells in the present study are consistent with these findings from previous studies, which offers a partial explanation for the concept of “detection of AGEs through noninvasive lenses to reflect kidney damage”. We believe that lens AGEs are closely related to those in the kidney.

AGEs contain substances with fluorescent properties, such as pentosidine and imidazoline. Thus, AGEs in the human body can be detected by special instruments without specific exogenous labeling.AF of the lens, skin tissue and serum can represent the blood glucose level in DM patients to a certain extent (23–28).Lens AF measurement is a noninvasive and rapid method that can be readily used in doctors’ offices that indirectly shows AGE accumulation in the natural lens of the eye, most commonly due to DM (28, 29). The results of Frederick Cahn et al. showed that lens autofluorescence levels were highly predictive of diabetes and that progressively higher fluorescence deviations were observed in prediabetes, type 2 diabetes, and type 1 diabetes (30).However, the adoption of noninvasive AGEs technology to detect and evaluate DKD has not been reported. AGEs have auto fluorescent properties. Detection instruments emitting light waves of 430-470 mm cause AGEs to autofluorescence, and this AF can be measured (31).Skincare products can affect the results of skin AGE measurement, and this effect cannot be eliminated by hand washing or alcohol wiping (28);however, the lens is less affected by external interference. Therefore, this study used a noninvasive method to measure lens AGE levels to quickly identify affected individuals, hoping to provide a theoretical basis for the use of this method for early DKD screening.

Recent studies have shown that noninvasive detection of lens or skin AGE fluorescence could predict incident T2DM (9, 10).Therefore, we speculated that using noninvasive detection of lens AGEs fluorescence can predict the occurrence of DKD. We noninvasively assessed the research subjects with the lens AGEscan fluorescence detector mentioned in the Methods section above. Our findings showed that AGEs accumulation may be an independent risk factor for DKD. After excluding interference from sex and age and taking 0.306 as the cutoff, the incidence of DKD in the group with high lens AGEs levels was approximately 2.739 times that in the group with low lens AGEs levels. Thus, noninvasive detection technology for crystalline AGEs has acceptable sensitivity and specificity for screening T2DM patients for DKD. Additionally, the optimal critical value of crystalline AGEs is 0.306, suggesting that crystalline AGEs levels greater than or equal to 0.306 are associated with an increased risk for DKD, and patients with these values require follow-up.

In addition, chronic low-grade inflammation also plays an important role in the pathophysiological process of DKD. We explored the predictive power of different inflammatory cell parameters on the occurrence and outcomes of DKD. Our study also showed that the peripheral blood NLR was significantly different between patients with and without DKD, and the NLR in the non-DKD group was lower than that in the DKD group. These findings are similar to those of Z. A. Öztürk and M. Verdoia, A. et al. (32, 33). Our results showed that lens AGE levels were positively correlated with NLR.

However, as this was a single-center study, the sample size was limited, and the included research subjects were mostly older. As a result, further studies including more patients and different age groups are required in the future. Our research group plans to follow up with the diabetic patients who participated in this study, continue to verify our hypothesis and further observe changes in lens AGEs levels to predict DKD outcomes.

In conclusion, we proved that AGEs are closely related to DKD from cell, animal, and human experiments. Furthermore, we found that AGEs are an independent risk factor for predicting the occurrence of DKD. These results suggest that noninvasive lens AGEs detection technology has certain clinical value in diagnosing whether T2DM patients have DKD. Our findings will contribute to the early screening of DKD in patientswith T2DM.

## Data Availability Statement

The datasets presented in this study can be found in online repositories. The names of the repository/repositories and accession number(s) can be found in the article/supplementary material.

## Ethics Statement

The studies involving human participants were reviewed and approved by Ethics Committee of Shanghai Fifth People’s Hospital Affiliated to Fudan University. The patients/participants provided their written informed consent to participate in this study. The animal study was reviewed and approved by Ethics Committee of Shanghai Fifth People’s Hospital Affiliated to Fudan University.

## Author Contributions

X-MZ,YG and M-XY contributed to conception and design of the study. YG, M-XY and X-DZ organized the database. YG performed the statistical analysis. X-MZ wrote the first draft of the manuscript. YG, M-XY, X-DZ, RZ, Y-YW, MZ, QY, Z-YY, JL, B-BZ, BY wrote sections of the manuscript. All authors contributed to manuscript revision, read, and approved the submitted version.

## Funding

The study was supported by the Shanghai Municipal Health Commission (No. 202140345) and the Medical Key Faculty Foundation of Shanghai (No. ZK2019B15).

## Conflict of Interest

The authors declare that the research was conducted in the absence of any commercial or financial relationships that could be construed as a potential conflict of interest.

## Publisher’s Note

All claims expressed in this article are solely those of the authors and do not necessarily represent those of their affiliated organizations, or those of the publisher, the editors and the reviewers. Any product that may be evaluated in this article, or claim that may be made by its manufacturer, is not guaranteed or endorsed by the publisher.
